# Assessing midwives' breastfeeding knowledge: Properties of the Newborn Feeding Ability questionnaire and Breastfeeding Initiation Practices scale

**DOI:** 10.1186/1746-4358-3-7

**Published:** 2008-04-30

**Authors:** Debra K Creedy, Ruth M Cantrill, Marie Cooke

**Affiliations:** 1 Research Centre for Clinical and Community Practice Innovation, Griffith University, Brisbane, Australia; 2 National University of Singapore, Singapore

## Abstract

**Background:**

There are few reliable and valid tools to assess lactation and infant feeding knowledge and practices. This study tested the psychometric properties of two new scales, the Newborn Feeding Ability (NFA) questionnaire and Breastfeeding Initiation Practices (BIP) scale to assess midwives' breastfeeding knowledge and practices specific to breastfeeding initiation.

**Methods:**

A national postal survey of Australian midwives (n = 3500) was conducted in October 2001. Reliability was determined through Cronbach's alpha coefficient and stability determined by a test-retest. Content validity was established through a critical review of literature and review by an expert panel. Construct validity was informed by an exploratory factor analysis and principle component analysis with varimax rotation. Correlations between NFA and BKQ knowledge subscale scores and BIP and BKQ practice subscale scores assessed criterion validity. A multiple hierarchical regression analysis determined predictive validity of the NFA and BIP.

**Results:**

A response rate of 31.6% (n = 1107) was achieved. Adequate internal consistency was established for both instruments. Five factors on the NFA questionnaire were congruent with knowledge about effects of skin-to-skin contact, physiological stability, newborn innate abilities, work practices and effective breastfeeding. The BIP revealed three factors related to observing pre-feeding behavior, mother/baby care and attachment and positioning practices. Predictive validity of knowledge was moderate (r = 0.481, p < 0.01) and contributed to 31.5% of variance in reported practice. Midwives with high knowledge scores were more likely to report best practice when assisting mothers to initiate breastfeeding. Midwives with more personal breastfeeding experience scored higher on all scales.

**Conclusion:**

The Newborn Feeding Ability questionnaire and the Breastfeeding Initiation Practices scale can contribute to practice development by assessing lactation and infant feeding knowledge and practice deficits. Individual learning needs can be identified, and effectiveness of education interventions evaluated using these tools. Further testing is required with other samples of midwives and health professionals involved in the promotion of breastfeeding.

## Background

Healthcare service providers play a key support and educative role to mothers about human lactation and infant feeding [1-4]. Despite this important role, health professional education about breastfeeding is generally lacking [4,5]. In recent years there has been renewed interest in health professionals' knowledge of lactation and infant feeding and materal satisfaction with the provision of breastfeeding support [1,6-9].

Accurate assessment of health professional breastfeeding knowledge can identify learning deficits, inform the content of breastfeeding education programs and improve practice [1,5,10,11]. Various studies have attempted to assess health professionals' knowledge and practice concerning lactation and infant feeding. Focus group discussions have explored women's perceptions of health professional support and revealed conflicting advice and poor practice [9,12-14]. Similarly, interviews with midwives, nurses and mothers [9,15-19] to determine relationships between health professional lactation knowledge and maternal satisfaction with service provision have identified knowledge and practice deficits, as have studies on support for breastfeeding initation and duration [20-22].

Using a pre-post test design, White, Simon and Bryan [20] found that nurse education about infant feeding behavior and cues had a positive impact on the mother-infant relationship. A recent survey of mothers found that perceptions of care and responsiveness towards their infants were enhanced when health professionals were knowledgeable about breastfeeding and provided continuity of care [22]. In another study, high breastfeeding knowledge scores and attitudes were predictive of supportive behavior by nurses in providing information and technical and emotional support to breastfeeding women [23]. Despite the positive findings of these studies, midwives' knowledge of breastfeeding and lactation management has been relatively neglected over many years [7,24]

Several tools have been used to assess breastfeeding knowledge amongst various health professional groups [23,25-31] but few authors have reported reliability and validity of such measures. Furthermore, there has been little emphasis on midwives' knowledge of the neurobehavioral adaptation of both mother and infant as the basis for effective breastfeeding care. This paper reports on the development of two new instruments, the Newborn Feeding Ability (NFA) questionnaire and Breastfeeding Initiation Practices (BIP) scale to assess midwives' breastfeeding knowledge and practice.

### A review of existing measures of breastfeeding knowledge and practice

Several tools have attempted to assess breastfeeding knowledge and practice but few have been independently evaluated. A widely used tool, the Breastfeeding Knowledge Questionnaire (BKQ) [25] assessed general breastfeeding knowledge and attitudes amongst 3275 US resident doctors and physicians in pediatrics, obstetrics, gynecology or family medicine. The BKQ has also been used to assess general breastfeeding knowledge and practices amongst North American nurse practitioners and nurse-midwives [27]. The original BKQ does not produce a total score and reliability has not been reported because response options on BKQ items include 5-point Likert scale items, yes/no responses, selecting an option and open-ended comment. Results are presented as percentage correct for each item, and chi square comparisons of professional characteristics and item responses. This scoring approach is time consuming, does not permit the calculation of a total score for comparisons across time and groups, nor allow statistical analysis of individual and group performance.

Other tools assessing breastfeeding knowledge have used predominantly open-ended questions [32] requiring a level of subjectivity in coding, and findings are open to interpretation. Some items on existing tools are now out-dated [31], assess a limited range of knowledge [25-27,31] or are administered under test conditions [30] that can be threatening to respondents and inhibit participation. Furthermore, existing tools examine basic knowledge about the health benefits of human milk, breastfeeding management and decision making for common breastfeeding problems. There is an assumption that this level of knowledge is sufficient for best practice [5]. Deeper level knowledge related to neonatal and maternal neurobehavioral adaptation for the initiation of breastfeeding is relatively ignored [4,33].

There is considerable evidence that newborn babies possess innate reflexes that enable them to find the nipple, attach correctly and breastfeed effectively, provided they are given the opportunity to remain in naked body contact (skin-to-skin) with their mother for a sufficient length of time [34-42]. Continuous skin-to-skin contact after birth until the infant actively takes the first breastfeed is a best practice standard and recommended for Baby Friendly Hospital Initiative (BFHI) accreditation [2,43]. If newborn babies' needs are understood and their innate abilities utilized at the first breastfeed, potential breastfeeding problems may be minimized and initiation rates and maternal satisfaction improved [35,44,45]. It may therefore be particularly important to assess midwives' deeper level knowledge and practices in managing the first breastfeed. This article reports on two new tools to measure breastfeeding knowledge and practices.

## Methods

### Aim

To test the psychometric properties of two new scales, the Newborn Feeding Ability (NFA) questionnaire and Breastfeeding Initiation Practices (BIP) scale that assess midwives' breastfeeding knowledge and practice. The existing BKQ [25] was revised and included to assess criterion validity of the NFA and BIP.

### Design

A descriptive survey design was used.

### Sample

A national sample of midwives was accessed through the Australian College of Midwives Inc (ACMI). Midwives, registered nurse-midwives and midwifery students in clinical practice who interact with women antenatally, during childbirth or in the immediate postnatal period were invited to participate. Nurses involved in maternity care who were not midwives were excluded as the study investigated midwives' knowledge and practice. A total of 1105 usable questionnaires (out of a possible 3,500) were returned, giving a response rate of 31.6%.

### Data Collection

An information letter, questionnaire and reply paid envelope were distributed through ACMI in their national newsletter in October 2001. Responses were anonymous and no reminder notices were sent.

### Measures

Personal and professional details of respondents were sought including years of experience as a midwife, education qualifications and personal breastfeeding experience. Commitment to continuing professional education was assessed by the number of breastfeeding learning activities/resources accessed in the previous 12 months.

#### Newborn Feeding Ability questionnaire

The Newborn Feeding Ability (NFA) questionnaire has 21 items with responses on a 5-point Likert scale of 1 = "strongly disagree" to 5 = "strongly agree". Items assess knowledge about 1) physiological and emotional benefits of skin-to-skin contact for newborns and mothers; 2) indicators of effective suckling and milk transfer; and 3) work practices that interfere with newborn feeding ability. Four items are reverse scored to minimize response bias. The NFA has a possible total score of 105 with higher scores reflecting greater knowledge (seen Additional file 1). Correct responses on NFA items and relevant references are displayed in Table 1.

**Table 1:** Correct responses to Newborn Feeding Ability items and evidence source

**Correct responses to items**	**Evidence**
1. A normal full term infant is born with instinctive reflex ability to breastfeed effectively	[40]
2. Newborns will develop predictable, coordinated feeding behaviors within minutes of birth	[38, 39]
3. Newborns can instinctively find the nipple without help and attach correctly to the breast	[35]
4. Newborns will be guided to the nipple by their sense of smell	[37]
5. Skin-to-skin contact is important to help stabilize newborn breathing	[58, 59]
6. A newborn's heart rate is stabilized by skin-to-skin contact	[60]
7. Skin-to-skin contact is important to prevent heat loss in newborn babies	[61, 62]
8. A newborn's blood sugar levels are stabilized by skin-to-skin contact	[62, 63]
9. Skin-to-skin contact helps the flow of colostrum after birth	[64]
10. Uninterrupted skin-to-skin contact immediately after birth is important for newborn breastfeeding performance	[35, 41]
11. A mother is more likely to accept and feel warm toward her baby if skin-to-skin contact happens immediately after birth	[64]
12. Hours of continuous skin-to-skin contact can help a newborn baby learn to feed	[34]
13. Midwives and mothers know the baby is getting colostrum at the first breastfeed when they hear the baby swallow	[65]
14 Midwives and mothers know the baby is getting colostrum at the first breastfeed when they see the baby swallow	[65]
15. Separation of a newborn from the mother at birth can cause harmful stress to the baby	[40, 66]
16. Birth trauma may interfere with the proper coordination of an infant's natural suckling reflexes	[67]
17. Interrupting skin-to-skin contact within 15–20 minutes of birth seriously disturbs the suckling reflexes for correct attachment	[35, 68]
18*. Immediately after birth, uninterrupted skin-to-skin contact should be facilitated until after the first breastfeed	[42, 43]
19*. Skin-to-skin contact to initiate feeding is of higher priority than wrapping the baby	[62]
20*. Skin-to-skin contact to breastfeed should take precedence over completion of required documentation	[44]
21*. Most mothers prefer to hold their baby immediately after birth rather than be cleaned	[44]

* reversed scored items

#### Breastfeeding Initiation Practice scale

The Breastfeeding Initiation Practice (BIP) scale has twelve items and uses a case scenario involving commonly observed birthing room events. "Chloe" is a 20 year old primipara who received narcotic analgesia during labour. Her mother, who was present at the birth, requested to know the baby's weight. Chloe planned to breastfeed, had no pregnancy risk factors, an uneventful spontaneous labour, and gave birth vaginally to a full-term gestation infant who was vigorous at birth. BIP items require respondents to report on the likelihood of the baby being able to find the nipple, attach and breastfeed effectively within the first hour of birth. These items are rated on a five point Likert scale of 1 = "unlikely" to 5 = "highly likely". The extent to which midwives facilitated uninterrupted skin-to-skin contact immediately after birth and promoted baby's innate ability to breastfeed are assessed on a scale of 1 = "never" to 5 = "always". Half the items are reverse scored with a possible total score of 60. Higher scores reflect better practice. The BIP can be seen in the Additional file.

#### Breastfeeding Knowledge Questionnaire

The Breastfeeding Knowledge Questionnaire (BKQ) [25] was used to assess criterion validity of the NFA and BIP. It assesses knowledge on benefits of human milk (7 items), advice health professionals may offer breastfeeding mothers on a range of common issues (6 items) and two items on practical management. The knowledge component of the BKQ was revised to incorporate a five point Likert scale of 1 = "strongly disagree" to 5 = "strongly agree". Items are summed to give a possible knowledge subscale score of 35 with higher scores reflecting better understanding. The practice advice component was revised to include a scale of 1 = "unsure", 2 = "never" to 4 = "always". Items are recoded so the correct answer of "never" yields the highest score of 2 and "unsure" is allocated a score of 1. All other responses are incorrect and do not attract a score. The six items are summed to give a possible subscale score of 12. Two items relate to management of breastfeeding in the case of a four day old, otherwise healthy, jaundiced baby, and maternal perceptions of milk inadequacy at two weeks. The correct response "to continue or encourage more frequent breastfeeding" is allocated 4 points for a total possible score of eight. The sum of responses gives an overall general breastfeeding knowledge and practice score of 55.

### Expert review

To enhance content validity, NFA and BIP items were informed by a critical review of the research literature and midwifery texts (as outlined in Table 1). Generated items and ideal answers were reviewed by an expert panel of eleven members consisting of six midwives (three of whom were International Board Certified Lactation Consultants), a researcher, educator and lactation consultant in private practice, a pediatrician involved in clinical research, a lactation consultant/medical scientist and a lactation consultant/speech therapist. There was 100% agreement amongst panel members on correct responses. The panel recommended minor adjustments to wording and structure of items for clarity.

### Pilot study

Fifteen midwives who worked across all clinical areas (antenatal, birth suite and postnatal ward) completed the draft survey and gave feedback in regards to level of difficulty, ambiguity of statements, relevance, repetition, as well as any other comments they wished to offer to improve the questionnaire. The survey was repeated after two weeks.

### Ethical considerations

The study received Griffith University Human Research Ethics Committee approval. Participants received written information about the purpose of the study and our intention to publish survey results. There were no anticipated physical, social or legal risks associated with participation. Informed consent was implied if participants completed and returned the questionnaire.

### Data coding and analysis

Data were coded and analysed using the Statistical Package for the Social Sciences (SPSS) version 12. Demographic details of respondents were analysed using descriptive statistics mean, standard deviation and range. Years of experience as a midwife were categorized into four groups of 1 – 5 years, 6 – 10 years, 11 – 15 years and > 15 years. Experience less than six years was categorized into three groups: midwifery students, new graduate midwives and midwives with 2 – 5 years of experience. Midwives' personal breastfeeding experience was categorized into two groups (those having breastfed an infant for three months or longer and participants with less than three months or no breastfeeding experience). Commitment to continuing professional education was assessed by the number of breastfeeding learning resources/activities accessed in the previous twelve months from a list of activities provided. These items included reading relevant journal articles, attending workplace breastfeeding seminars and conference attendance. Respondents were categorised into three groups of low (0 – 5 activities), moderate (6 – 7 activities) and high (8 – 12 activities each year) commitment. Continuing professional education results are reported elsewhere [46].

Total and subscale scores were derived for the NFA, BIP and revised BKQ. Cases with missing values were excluded from analysis. Deletion of cases with missing data (less than 5%) that are a random sub-sample of the whole set is likely to yield similar results to any other method of handling missing values [47]. A bivariate Pearson product-moment correlation was conducted between all continuous variables. Associations between interval and nominal variables were assessed using Spearman's rank order correlation. Differences among group means were determined using ANOVA and Independent Samples T-test. Reliability was determined through Cronbach's alpha coefficient and stability determined by a test-retest. Construct validity was informed by an exploratory factor analysis and principle component analysis with varimax rotation. Correlations between NFA and BKQ knowledge subscale scores and BIP and BKQ practice subscale scores assessed criterion validity. A multiple hierarchical regression analysis was used to determine predictive validity of the NFA and BIP.

## Results

A total of 1107 questionnaires (31.6%) were returned. This is a reasonable response rate for an anonymous postal survey [48]. Participant characteristics and scores on the BKQ and NFA have been published elsewhere [28,29]. In summary, the mean age of participants was 41 years (range of 23 to 67 years, SD = 8.39). The average length of midwifery experience was 12.7 years (range 0 to 40 years, SD = 8.4 years). Over two thirds of respondents or their partner (to allow for male midwives) had breastfed an infant (69.6%, n = 771) or breastfed for longer than three months (65.9 %, n = 729). Most participants (73.1%, n = 810) received their original professional midwifery education in hospital-based certificate courses. A proportion of midwives (17.5%, n = 194) were accredited with the International Board of Lactation Consultant Examiners (IBLCE). Around 39% (n = 403) of participants had between six and fifteen years of midwifery experience, 36% (n = 398) were experienced for over fifteen years and about 19% had between two and five years of experience. See Table 2 for sample characteristics.

**Table 2:** Characteristics of sample

	**Sample n (%)**
**TOTAL**	**1105 (100)**

**Age**	
20–30	127 (11.5)
31–40	385 (34.8)
41–50	416 (37.7)
Over 50	150 (13.6)
Missing data	27 (2.4)
**Sex**	
Female	1080 (97.7)
Male	15 (1.4)
Missing data	10 (0.9)
**Years of experience**	
0 years (student midwives)	15 (1.4)
1 year (new graduate midwives)	51 (4.6)
2 – 5 years	208 (18.8)
6 – 10 years	232 (21.0)
11 – 15 years	198 (17.9)
Over 15 years	396 (35.8)
Missing	5 (0.5)
**Midwifery qualification**	
Hospital	808 (73.0)
University	284 (25.7)
Direct entry	7 (0.7)
Missing data/Nurse (non midwife)	6 (0.6)
**IBCLC accredited**	
Yes	194 (17.6)
No	908 (82.1)
Missing data	3 (0.3)
**Breastfed > 3 months**	
Yes	729 (66)
No	365 (33)
Missing data	11 (1.0)

Mean score for the NFA was 85.94 out of 105 (range 40 – 105, SD = 10.55) and total mean BIP score was 46.84 out of 60 (range 25 – 57, SD = 4.68). Mean BKQ score was 48.08 out of 55 (range 16 – 55, SD = 5.47). A secondary analysis of BKQ data for the purposes of the present paper revealed a mean knowledge subscale score of 30.57 out of 35 (range 9 – 35, SD = 3.58), and a mean practice subscale score of 17.57 out of 20 (range 0 – 21, SD = 3.05).

### Reliability

Cronbach's alpha coefficients demonstrate adequate internal consistency [48] for the NFA (α = 0.87) and BIP (α = 0.74). Internal reliability for the BKQ was 0.78. There was no correlation between test-retest scores of the pilot group at eight weeks (r = 0.345, p = 0.104). This could be explained by the small number of participants in the pilot study (n = 15) and an expectation of change on a knowledge test.

### Construct validity

To summarise patterns of correlations among items and determine plausible underlying structures of the NFA, BIP and BKQ, exploratory factor analyses were conducted. NFA analysis revealed five factors with eigenvalues greater than one that explained 59% of variance (as outlined in Table 3). Factor 1 had an eigenvalue of 6.84, explained 32.5% of variance and was congruent with knowledge of skin-to-skin contact effects. The other four factors were "physiological stability" associated with skin-to-skin contact (eigenvalue = 1.84), "innate ability" of the infant to suckle effectively (eigenvalue = 1.34), work practices that enhance the innate abilities of neonates (eigenvalue = 1.27) and elements of effective breastfeeding (eigenvalue = 1.11).

**Table 3:** Newborn Feeding Ability with Principle Component Varimax rotation

	**Component**				
	**1 Skin contact effects**	**2 Physiological stability**	**3 Innate ability**	**4 Work practices**	**5 Effective breastfeeding**
Variance Explained	32.55%	8.78%	6.46%	6.05%	5.28%

**Factor 1 Knowledge Skin contact effects**					
11. A mother is more likely to accept and feel warm toward her baby if skin-to-skin contact happens immediately after birth	0.711				
15. Separation of a newborn from the mother at birth can cause harmful stress to the baby	0.692				
10. Uninterrupted skin-to-skin contact immediately after birth is important for newborn breastfeeding performance	0.667				
16. Birth trauma may interfere with the proper coordination of an infant's natural suckling reflexes	0.662				
17. Interrupting skin-to-skin contact within 15–20 minutes of birth seriously disturbs the suckling reflexes for correct attachment	0.632				
12. Hours of continuous skin-to-skin contact can help a newborn baby learn to feed	0.617				
7. Skin-to-skin contact is important to prevent heat loss in newborn babies	0.425				
					
**Factor 2 Physiological stability**					
6. A newborn's heart rate is stabilized by skin-to-skin contact		0.821			
5. Skin-to-skin contact is important to help stabilize newborn breathing		0.817			
8. A newborn's blood sugar levels are stabilized by skin-to-skin contact		0.752			
9. Skin-to-skin contact helps the flow of colostrum after birth		0.457			
					
**Factor 3 Innate ability**					
2. Newborns will develop predictable, coordinated feeding behaviors within minutes of birth			0.764		
3. Newborns can instinctively find the nipple without help and attach correctly to the breast			0.748		
4. Newborns will be guided to the nipple by their sense of smell			0.703		
1. A normal full term infant is born with instinctive reflex ability to breastfeed effectively			0.625		
					
**Factor 4 Work practices**					
20. Time required for skin-to-skin contact to breastfeed interferes with completion of required documentation				0.784	
18. There is no time immediately after birth to allow uninterrupted skin-to-skin contact until the first breastfeed				0.711	
21. Most mothers want to be cleaned up immediately after birth rather than hold their baby				0.661	
19. Prevention of heat loss by wrapping the baby is of higher priority than skin-to-skin contact to initiate feeding behaviours.				0.655	
					
**Factor 5 Effective breastfeeding**					
13. Midwives and mothers know the baby is getting colostrum at the first breastfeed when they hear the baby swallow					0.846
14 Midwives and mothers know the baby is getting colostrum at the first breastfeed when they see the baby swallow					0.816

Cronbach Alpha	0.84	0.83	0.76	0.70	0.56

Analysis of BIP revealed three factors with eigenvalues greater than one explaining 49.9% of variance. Factor 1 which had an eigenvalue of 3.22, explained 26.8% of variance and was congruent with practice of observing pre-feeding behavior. Factor 2 (eigenvalue = 1.60) reflected practices associated with mother/baby care and the third factor (eigenvalue = 1.16) was related to attachment and positioning practices (see Table 4).

**Table 4:** Breastfeeding Initiation Practices with Principle Component Analysis Varimax rotation

	**Component**		
	**1 Attention to feeding**	**2 Care of baby**	**3 Assist attach**
Variance Explained	26.84%	13.34%	9.67%
			
**Factor 1 Attention to feeding**			
11. Ask Chloe what she would like to do and explain the natural feeding ability of a newborn	0.737		
9. Teach Chloe how to position and attach baby for optimal breastfeeding	0.703		
10. Encourage Chloe to take time to allow the baby to self attach with minimal assistance and explain a newborn's natural ability to breastfeed	0.643		
7. Encourage Chloe and the family to watch for signs of baby's readiness to feed	0.632		
			
**Factor 2 Care of baby**			
4. Dry and wrap the baby before giving to the parents		0.723	
6. Place the baby under a radiant heater for assessment, weighting and measuring before the first breastfeed attempt		0.618	
3. Help Chloe hold her naked baby in skin-to-skin		0.582	
			
12. Wait until Chloe is showered and able to sit up comfortably before offering assistance		0.559	
5. Place baby in skin-to-skin on Chloe's chest, dry the baby and cover with a warm towel		0.557	
2. Routinely suction the baby at birth before giving to Chloe		0.513	
			
**Factor 3 Assist attach**			
8. "Put the baby on" the breast for her			0.785
1. Chloe's baby is likely to attach correctly to the breast without assistance within the first hour of birth			0.480

Cronbach Alpha	0.68	0.70	0.29

Principle component analysis with Varimax rotation of thirteen questions suitable for analysis in the BKQ revealed two components with eigenvalues exceeding 1 explaining 47.8% of variance (see Table 5). Factor 1, "general breastfeeding knowledge" (eigenvalue = 3.55) explained 23.7% of variance. Factor 2, "practice advice" (eigenvalue = 2.66) explained 17.7% of variance. The analysis suggests the BKQ knowledge and practice subscales are useful but revision of items may strengthen the practice subscale.

**Table 5:** Breastfeeding Knowledge Questionnaire with Principle Component Analysis Varimax rotation 2 factor solution

	**Component**	
	**1 Breastfeeding protection**	**2 Practice Advice**
Variance Explained	27.5%	19.7%
		
**Factor 1 Breastfeeding protection**		
5. Breastfeeding decreases the incidence of gastroenteritis	0.852	
6. Breastfeeding provides increased immune function	0.820	
4. Breastfeeding protects against allergic response to protein food allergy	0.808	
3. Breastfeeding decreases the incidence of otitis media	0.761	
2. Exclusive breastfeeding (without supplementation) is the most beneficial form of nutrition for the first six months of an infant's life	0.685	
7. Supplementing breastfeeding with formula during the first two weeks of life is a cause of breastfeeding failure	0.451	
1. Counseling by midwives is effective in encouraging more women to breastfeed	0.444	
		
**Factor 2 Practice Advice**		
13. If baby does not seem satisfied tell the mother to stop breastfeeding completely		0.720
11. If baby is teething tell the mother to stop breastfeeding completely		0.661
9. If a mother has insufficient milk supply tell her to stop breastfeeding completely		0.652
12. If baby has frequent, loose stools tell the mother to stop breastfeeding completely		0.644
8. If a mother has mastitis tell her to stop breastfeeding completely		0.559
10. If mother has a breast abscess tell her to stop breastfeeding completely		0.543

Cronbach Alpha	0.85*	0.69

* Alpha with Items 7 & 1 removed.

### Criterion validity

A correlation between NFA and BKQ knowledge subscale score (r = 0.578, p = 0.001) was found. There was a correlation between BIP and BKQ practice subscale score (r = 0.221, p = 0.001). Content validity was enhanced as BKQ items were based on previous studies of medical physicians', midwives' and nurses' breastfeeding knowledge [25,27], while NFA and BIP items were drawn from a critical review of literature on breastfeeding initiation and expert review.

### Predictive validity

NFA scores contributed to 31.5% of variance in reported practice (F (1, 1061) = 504.6, β = 0.56, R^2 ^= 0.314; p = 0.001) indicating moderate predictive validity. Midwives with more knowledge of newborn feeding ability were more likely to report best practices when assisting mothers to initiate breastfeeding [29].

### Group comparisons

The present study examined the extent to which knowledge and practices were associated with professional and personal characteristics including years of experience, commitment to professional development and personal breastfeeding experience. A known group analysis (years of clinical experience and personal breastfeeding experience) was conducted and significant differences in NFA and BIP scores were found between groups.

#### Clinical experience

Years of clinical experience were categorized into four groups (excluding student midwives) and found to be associated with higher NFA scores (F (3, 1021) = 3.29, p = 0.02). Post-hoc comparisons using the Tukey HSD statistic revealed midwives with 11 – 15 years experience scored significantly higher (mean = 87.34, SD = 10.19) on the NFA than colleagues with less than six years experience (mean = 84.34, SD = 9.55). Mean NFA scores of the groups with 6–10 years experience (mean = 86.14, SD = 10.56) and midwives with over 15 years experience (mean = 86.37, SD = 11.26) did not differ significantly from other groups. Participants with more than 15 and 11 – 15 years clinical experience achieved significantly higher BIP scores (mean = 47.45, SD = 4.39; M = 47.23, SD = 5.06) than less experienced colleagues of 6 to 10 years (mean = 46.34, SD = 4.31) and 1 to 5 years of experience (mean = 46.03, SD = 4.83).

#### New graduate and student midwives

A small number of midwifery students (n = 15) were included in the study analysis and categorised as 0 years experience. There were no statistically significant differences in NFA scores within the six groups of participants experienced for 0 – 5 years (F (5, 264) = 0.322, p = 0.9). While NFA mean scores for students (mean = 82.1, SD = 8.34) were lower than midwives of 1, 2, 3, 4 and 5 years of experience, the mean scores for midwives of 5 years of experience declined in comparison to other years and were closer to student level (mean = 83.7, SD = 9.78). Likewise no significant diferences were detected in BIP scores between students and midwives with one or more years and up to five years of experience. Mean scores of BIP for midwifery students (mean = 45.07, SD = 4.6.1) were close to those of midwives with five years of experience (mean = 45.05, SD = 5.20).

When students and new graduate midwives were pooled, no significant differences were found in NFA scores between the student/new graduate group and midwives with 2 to 5 years experience nor were BIP scores significantly different between the two groups. Interestingly, the mean score for the student/new graduate group (mean = 46.05, SD = 4.35) was slightly higher than midwives with 2 – 5 years experience (mean = 45.96, SD = SD 4.91), but the difference was not statistically significant. The BKQ scores between the two groups did not differ significantly although it did approach significance (t (1, 269) = 1.94, p = 0.053).

#### Professional development

Commitment to continuing professional development was associated with higher NFA (r = 0.236, p = 0.001) and BIP (r = 0.147, p = 0.001) scores. Differences between groups were found in mean scores for the NFA (F (2, 1058) = 29.248, p = 0.001) and BIP (F (2, 1046) = 12.425, p = 0.001). Post-hoc comparisons identified significant differences on NFA scores for participants who reported high commitment (mean = 89.7, SD = 10.17) compared with colleagues with moderate (mean = 85.65, SD = 10.16), or low (mean = 83.27, SD = 10.49) commitment.

#### Personal breastfeeding experience

Few studies have reported the influence of personal breastfeeding experience on professional knowledge and practice. In the present study, personal breastfeeding experience was associated with higher NFA (r = 0.072, p = 0.02) and BIP (r = 0.088, p = 0.004) scores. Midwives with at least three months personal breastfeeding experience obtained significantly higher mean scores on the BKQ knowledge subscale (t = 2.629, p = 0.009) and BIP (t (1, 1037) = 2.849, p = 0.004) than colleagues with less or no breastfeeding experience. Differences between the two groups for NFA mean scores were not significant, suggesting that personal breastfeeding experience is not associated with in-depth knowledge of newborn innate feeding abilities.

## Discussion

The present results need to be considered in light of limitations. Although a thirty-percent response rate is acceptable for an anonymous postal survey it could be that midwives most interested in breastfeeding promotion responded and their views may differ from non-respondents. Self-report instruments about practice highlight what respondents say they do but may not reflect actual practice. We attempted to minimise this risk by assuring anonymity and requesting complete honesty, but future research should include an observation study of practice. The three instruments were separated in the survey form but may have engendered a certain response set. Different responses may have been achieved if the instruments had been distributed individually. To confirm validity, the instruments need to be tested with other health professional populations.

This study provides initial support for the reliability and validity of the NFA and BIP scales. Factor analysis of the revised BKQ confirmed its focus on knowledge and practice and will enhance its use in health professional education and practice. Unlike previous measures, the NFA assesses in-depth knowledge with regards to benefits of continuous skin-to-skin contact between mother and baby for physiological stability and coordinated attachment. The BIP assesses practice for breastfeeding initiation. Accurate assessment of health professional knowledge and practice for the initiation of breastfeeding using standardised measures can help to identify learning deficits, inform the content of educational interventions and enhance the likelihood of best practice in the workplace [5,6,8,49]. The development of accurate assessment tools is important in light of on-going concerns about health professional breastfeeding knowledge and practice [1,6,18,24,25,27,28,50,51].

Further testing and refinement of the NFA and BIP is warranted. We recommend the inclusion of more items on assessment of milk transfer and assessing effective breastfeeding to enhance internal reliability of that subscale. Items regarding the effects of analgesia on newborn feeding ability need to be increased in number and refined. Emphasizing the adverse effects of narcotic analgesia administered to the mother during birth on newborn feeding abilities may influence midwives' knowledge of these which in turn can impact on practice and outcomes for mothers and babies. Perhaps further items regarding knowledge of suckling ability of premature and the 'near term infant' could be expanded in future questionnaires to enhance content.

Associations between midwives' characteristics, knowledge and reported practice were interesting. Midwives with 11 to 15 years clinical experience scored more highly in both general breastfeeding (BKQ) and in-depth (NFA) knowledge than colleagues with less than six years experience. Although not significant, groups with 6 – 10 years and over 15 years clinical experience also scored less than the group with 11 to 15 years experience. However, significantly higher practice scores were reported by groups with over 15 years and 11 to 15 years of clinical experience compared with groups with at least 10 years experience. A decrease in breastfeeding knowledge amongst health professionals including midwives with advancing years since their initial education has been reported previously [52]. As suggested by Lowe [52], the comparative decline in knowledge of health professionals with more years of clinical experience could be attributed to a lack of participation in breastfeeding education as part of their continuing professional development. This rationale is highly probable considering recent interest and discussions regarding ongoing difficulties to improve the uptake of available lactation and infant feeding professional development education and resources for midwives and other health professionals [6]. In Australia, continuing education specifically to update breastfeeding knowledge is mainly taken up by those hospitals moving toward BFHI accreditation.

Higher practice scores demonstrated by more experienced midwives could be a result of those midwives understanding what works well in practice without knowing the scientific basis of continuous skin-to-skin contact and newborn innate feeding abilities. On the other hand, midwives with more experience may be committed to professional development. For instance, almost half the midwives with International Board Certified Lactation Consultant (IBCLC) certification, participating in the study reported over 15 years of clinical midwifery experience. The potential incongruence between knowledge and practice needs further investigation. Consumers consistently report conflicting advice by health professionals with regards to breastfeeding issues higlighting the need for efficient assessment of learning needs and education [12,18,53,54]. Greater emphasis on the practical application of breastfeeding knowledge amongst midwives to enhance consistency of breastfeeding advice and support to mothers has been recommended [5,7,8,10].

The tools used in the present study focus on a specialised area of knowledge and could be used to identify knowledge and practice deficits in the area of newborn feeding behavior for breastfeeding initiation. This was not an included area of knowledge on a recent education needs analysis conducted by Wallace and Kosmala-Anderson [4] but could be added as an important area of knowledge for inclusion in education programs on lactation and infant feeding.

As expected, midwives who were committed to their own professional development scored highly on both measures of breastfeeding knowledge and practice. Results of the present study indicate that midwives who keep abreast of advancing knowledge have a better understanding and application of research evidence than those who do not access such resources. Hospitals need to make research-based resources readily available, encourage a learning culture, and provide easily accessible learning programs including computer based learning to refresh knowledge and inform practice [4,5].

Practice standards recommend that all health professionals providing care to mothers and/or infants complete at least 18 hours of education on human lactation and infant feeding [2]. Although there are a range of BFHI-based courses available [1] not all are used to meet the education needs of qualified practitioners. More efficient, cost-effective education methods need to be sought to enable employees to take professional responsibility for maintaining and improving their knowledge and practice [1,4,6,8].

Few studies have measured the influence of personal breastfeeding experience on knowledge and practice even though it is often suggested that health professionals rely on personal experience to inform practice [23,25,26,30,50,51]. In the present study, midwives with personal breastfeeding experience of more than three months scored higher across all measures than midwives with less or no breastfeeding experience. Lowe [52] reported that midwives who had difficult personal breastfeeding experiences were less knowledgeable about lactation and infant feeding matters. It could be that midwives with previous successful breastfeeding experience take a keen interest in supporting other women to breastfeed and keep their knowledge and practice updated.

While midwives' education can positively affect their personal breastfeeding experience [55], it cannot be assumed that personal breastfeeding experience provides sufficient knowledge in a professional capacity to adequately inform and support mothers [6,51,56]. Likewise midwifery colleagues cannot assume that professional education and experience is adequate for personal breastfeeding success [55]. It is up to midwives, other health professionals and education providers to ensure information from evidence based research is implemented for the care of women and their families at the time of breastfeeding initiation [6,10].

NFA scores demonstrated moderate predictive validity for practice. Continuing professional education activities should aim to address knowledge deficits and also measure practice outcomes. Maintaining the knowledge base of a high proportion of staff (80%) is essential for Baby Friendly Hospital accreditation [2,57]. The NFA and BIP could be useful tools in accreditation processes to assess knowledge and practice, inform the content and scope of continuing professional education activities, provide evidence of competence, and offer cost efficiencies by targeting specific deficits rather than offering lengthy, broad program content.

## Conclusion

Midwives involved in the care of women in the early postnatal period need a high level of knowledge concerning the benefits of breastmilk and management of common breastfeeding problems. In contrast to existing tools that measure midwives' basic knowledge and benefits of breastfeeding, the NFA and BIP were found to be reliable and valid tools for the assessment of knowledge of newborn feeding ability and reported breastfeeding initiation practices. Assessment of midwives' understanding of the neurobehavioral adaptation of both mother and infant supports the goals of BFHI [2]. Midwives require advanced knowledge and skill to optimise infants' use of their innate feeding ability to initiate breastfeeding. The tools will be useful in identifying knowledge and practice deficits, enabling education on lactation and infant feeding to target these deficits, and for enhancing evidence-based practice in the longer term.

## Competing interests

The authors declare that they have no competing interests.

## Authors' contributions

DC contributed to the design of the study, provided oversight of the project and drafted the manuscript. RC designed the NFA and BIP questionnaires, carried out the study, conducted data analysis and helped to draft and revise the manuscript. MC participated in the design and coordination of the study and revised the manuscript. All authors read and approved the final manuscript.

## Supplementary Material

Additional file 1 Newborn Feeding Ability (NFA) Questionnaire and Breastfeeding Initiation Practices (BIP) Scale. The NFA 21 item questionnaire using a 5-point Likert scale asking participant opinion on 1) benefits of skin-to-skin contact between mother and newborn, 2) indicators of effective suckling, 3) practices that interfere with newborn feeding ability. A case scenario and BIP 12 items rated on a 5-point Likert scale asking respondents to report the likelihood of baby being able to find the nipple and feed effectively. Click here for file
