# Supplementary material for: Assessing midwives' breastfeeding knowledge: Properties of the Newborn Feeding Ability questionnaire and Breastfeeding Initiation Practices scale
Source: Int Breastfeed J. 2008 Apr 30;3:7. doi: 10.1186/1746-4358-3-7 (PMC2396602; doi:10.1186/1746-4358-3-7)
Supplement: Additional file 1 — Newborn Feeding Ability (NFA) Questionnaire and Breastfeeding Initiation Practices (BIP) Scale. The NFA 21 item questionnaire using a 5-point Likert scale asking participant opinion on 1) benefits of skin-to-skin contact between mother and newborn, 2) indicators of effective suckling, 3) practices that interfere with newborn feeding ability. A case scenario and BIP 12 items rated on a 5-point Likert scale asking respondents to report the likelihood of baby being able to find the nipple and feed effectively. [file 1746-4358-3-7-S1.doc]

**Newborn Feeding Ability Questionnaire**

This questionnaire asks about newborn feeding ability.

*Please circle the number*  🄋  *beside your answer.*

What is your opinion regarding the following statement about newborn suckling ability

**1** A normal full term infant is born with instinctive reflex ability to breastfeed effectively?

1 strongly disagree 2 disagree 3 not sure 4 agree 5 strongly agree

A healthy newborn baby *(who is not sedated by any drugs)* kept in continuous skin-to-skin contact with the mother immediately after birth,

**2** Will develop predictable, coordinated feeding behaviors within minutes of birth

**1** strongly disagree **2** disagree **3** not sure **4** agree **5** strongly agree

**3** Can instinctively find the nipple without help and attach correctly to the breast

**1** strongly disagree  **2** disagree **3** not sure **4** agree **5** strongly agree

**4** Will be guided to the nipple by their sense of smell

**1** strongly disagree  **2** disagree **3** not sure **4**agree **5** strongly agree

What is your opinion regarding the benefits of continuous skin-to-skin contact for newborn babies and their mother?

**5** Skin–to-skin contact is important to help stabilize newborn breathing

**1** strongly disagree **2** disagree **3** not sure **4** agree **5** strongly agree

**6** A newborn’s heart rate is stabilized by skin–to-skin contact

**1** strongly disagree **2** disagree **3** not sure **4** agree **5** strongly agree

**7** Skin–to-skin contact is important to prevent heat loss in newborn babies

**1** strongly disagree **2** disagree  **3** not sure **4** agree  **5** strongly agree

**8** A newborn’s blood sugar levels are stabilized by skin–to-skin contact

**1** strongly disagree **2** disagree **3** not sure **4** agree **5** strongly agree

**9** Skin-to skin contact helps the flow of colostrum after birth

**1** strongly disagree **2** disagree **3** not sure **4** agree **5** strongly agree

**10** Uninterrupted skin-to-skin contact immediately after birth is important for newborn breastfeeding performance

**1** strongly disagree **2** disagree **3** not sure **4** agree **5** strongly agree

**11** A mother is more likely to accept and feel warm toward her baby if skin-to skin contact happens immediately after birth

**1** strongly disagree **2** disagree **3** not sure **4** agree **5** strongly agree

**12** Hours of continuous skin-to-skin contact can help a newborn baby learn to feed

**1** strongly disagree **2** disagree **3** not sure **4** agree  **5** strongly agree

To know the baby is getting colostrum at the first breastfeed, it is important that:

**13** Midwives and mothers can hear the baby swallowing colostrum

**1** strongly disagree  **2** disagree  **3** not sure **4** agree **5** strongly agree

**14** Midwives and mothers can see the baby swallowing

**1** strongly disagree **2** disagree  **3** not sure **4** agree **5** strongly agree

What is your opinion regarding the statement that:

15 Separation of a newborn from the mother at birth can cause harmful stress to the baby

1 strongly disagree 2 disagree 3 not sure 4 agree 5 strongly agree

16 Birth trauma may interfere with the proper coordination of an infant’s natural sucking reflexes

1 strongly disagree 2 disagree 3 not sure 4 agree 5 strongly agree

17 Interrupting skin-to-skin contact within 15-20 minutes of delivery seriously disturbs the suckling reflexes for correct attachment

1 strongly disagree 2 disagree 3 not sure 4 agree 5 strongly agree

18 *There is no time immediately after delivery to allow uninterrupted skin-to-skin contact until the first breastfeed

1 strongly disagree 2 disagree 3 not sure 4 agree 5 strongly agree

19 *Prevention of heat loss by wrapping the baby is of higher priority than skin-to-skin contact to initiate feeding behaviors.

1 strongly disagree 2 disagree 3 not sure 4 agree 5 strongly agree

20 *Time required for skin-to-skin contact to breastfeed interferes with completion of required legal documentation

1 strongly disagree 2 disagree 3 not sure 4 agree 5 strongly agree

21 *Most mothers want to be cleaned up immediately after delivery rather than hold their baby

1 strongly disagree 2 disagree 3 not sure 4 agree 5 strongly agree

**Breastfeeding Initiation Practice Scale**

This questionnaire presents a scenario and asks about your practice assisting women with the first breastfeed. You are the midwife attending the woman, at the time of initiation of the first breastfeed.

Please answer the following questions (circle the number 🄋 beside YOUR answer
OR write your answer in the space ______ provided)

SCENARIO

Chloe is a 20 year old 38 week gestation primipara.

Antenatally well, attended antenatal classes, plans to breastfeed.

Uneventful 10 hour labor, given pethidine 100 mg IMI 3 hours prior to birth.

Spontaneous Vertex Delivery (SVD) of a live healthy female infant Apgars 8:9,

weight 3320 grams requiring no medical intervention. Intact perineum.

Chloe’s mother is keen to find out how much the baby weighs.

Parents consented to routine newborn vitamin K and hepatitis B injections for baby.

**22** How would you view the likelihood of Chloe’s baby attaching correctly to the breast without assistance within the first hour of birth

**1** most unlikely **2** unlikely **3** likely **4** quite likely **5** highly likely

Provided no medical intervention was needed for Chloe or her baby, in this situation, I would:

**23** *Routinely suction the baby at birth before giving to Chloe

**1** never **2** occasionally **3** sometimes **4** mostly **5** always

**24** Help Chloe hold her naked baby skin-to-skin

**1** never **2** occasionally **3** sometimes **4** mostly **5** always

**25** *Dry and wrap the baby before giving to the parents

**1** never **2** occasionally **3** sometimes **4** mostly **5** always

**26** Place baby skin-to-skin on Chloe’s chest, dry the baby and cover with a warm towel

**1** never **2** occasionally **3** sometimes **4** mostly **5** always

**27** *Place the baby under a radiant heater for assessment, weighting and measuring before the first breastfeed attempt

**1** never **2** occasionally **3** sometimes **4** mostly **5** always

**28** Encourage Chloe and the family to watch for signs of baby’s readiness to feed

**1** never **2** occasionally **3** sometimes **4** mostly **5** always

**29** Other (*what would you do?*

**__________________________________________________________________________**

**__________________________________________________________________________**

To assist Chloe with the first breastfeed I would:

**30** *“Put the baby on” the breast for her

**1** never **2** occasionally **3** sometimes **4** mostly **5** always

**31** *Teach Chloe how to position and attach baby for optimal breastfeeding

**1** never **2** occasionally **3** sometimes **4** mostly **5** always

**32** Encourage Chloe to take time to allow the baby to self attach with minimal assistance and explain a newborn’s natural ability to breastfeed

**1** never **2** occasionally **3** sometimes **4** mostly **5** always

**33** Ask Chloe what she would like to do and explain the natural feeding ability of a newborn

**1** never **2** occasionally **3** sometimes **4** mostly **5** always

**34** *Wait until Chloe is showered and able to sit up comfortably before offering assistance

**1** never **2** occasionally **3** sometimes **4** mostly **5** always

**35** Other *(what would you do?)*

**__________________________________________________________________________**

**__________________________________________________________________________**

* Reverse scored items
